# Ochronotic Arthropathy and Alkaptonuria (Ochronosis): Case Report

**DOI:** 10.1055/s-0044-1779309

**Published:** 2024-04-23

**Authors:** Diego Ariel de Lima, Yaly Rebouças Carneiro Bastos, Jailson Castro de Aquino Filho, Danilo Lopes de Paiva, Renata Clazzer, Lana Lacerda de Lima

**Affiliations:** 1 Departamento de Ciências da Saúde, Centro de Ciências Biológicas e da Saúde (CCBS), Universidade Federal Rural do Semi-Árido (Ufersa), Mossoró, RN, Brasil; 2 Departamento de Ortopedia e Traumatologia, Hospital Regional Tarcísio Maia (HRTM), Mossoró, RN, Brasil; 3 Departamento de Ortopedia e Traumatologia, Hospital Otávio de Freitas (HOF), Recife, PE, Brasil

**Keywords:** alkaptonuria, arthropathy, ochronosis

## Abstract

Alkaptonuria (AKU) is a rare genetic condition resulting from a deficiency in the homogentisic acid oxidase enzyme, which is produced by the liver and kidneys, that interferes with the metabolism of the amino acids phenylalanine and tyrosine. Although it may not cause symptoms, AKU can lead to ochronosis, the abnormal accumulation in body tissues of a pigment called alkapton. Over time, this pigment accumulation in the joints may result in secondary osteoarthritis known as ochronotic arthropathy, the most debilitating form of the disease. Since this is a rare condition, not widely discussed, we herein report a case to describe a diagnosis of ochronotic arthropathy of the knee only identified during surgery. Given the rarity of this condition, especially in Brazil, case descriptions will help understand the national epidemiology and disseminate more information about alkaptonuria and its clinical manifestations, particularly those of osteoarticular nature.

## Introduction

Alkaptonuria (AKU) is a rare and autosomal recessive genetic condition resulting from a deficiency in the homogentisic acid oxidase enzyme, which is produced by the liver and kidneys, and plays a role in the metabolism of the amino acids phenylalanine and tyrosine. 1

The disease may be asymptomatic or lead to the development of ochronosis, a condition characterized by the accumulation of a homogentisic acid polymer, a pigment called alkapton, in body tissues. 2 About half of AKU carriers develop ochronosis. Most patients are men, at a rate of 2:1, older than 40 years of age. 2

Ochronosis can present with several significant clinical manifestations, including arthropathy, eye and skin pigmentation, dark urine (homogentisic aciduria), and cardiovascular involvement. Throughout the years, the accumulation of homogentisic acid pigments in the joints can cause secondary osteoarthritis, known as ochronotic arthropathy, the most disabling form of the disease. 2 3

Typically, AKU diagnosis relies on clinical findings, with subsequent confirmation by the detection of homogentisic acid in the urine at levels of up to 5 g in 24 hours. 2

Due to the rarity of this condition, especially in Brazil, case descriptions will provide insight into the national epidemiology and disseminate knowledge about AKU and its clinical manifestations, especially those of osteoarticular nature.

## Case Report



**Video 1** Knee arthroscopy showing advanced tricompartmental chondropathy, with dark coloration suggesting intra-articular pigment deposits (ochronotic arthropathy).


**Video 2** Knee arthroscopy showing advanced tricompartmental chondropathy, with dark coloration suggesting intra-articular pigment deposits (ochronotic arthropathy).


**Fig. 1:**
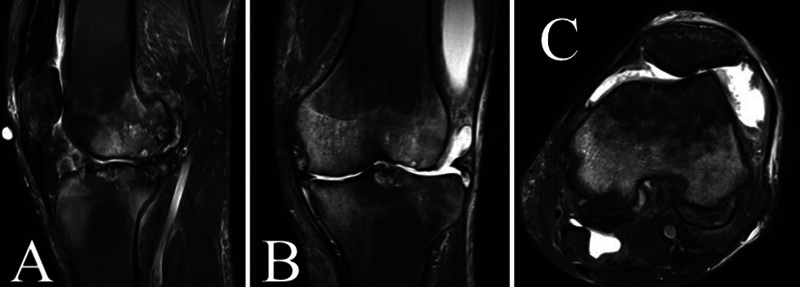
Magnetic resonance imaging of the left knee. ( **A** ) Sagittal section; ( **B** ) coronal section; ( **C** ) axial section.

A 46-year-old male patient presented to the Orthopedics Service complaining of bilateral knee pain and instability. The subject presented preserved lower limb alignment, positive results on the anterior drawer and pivot-shift tests, pain during joint interline palpation, and clinical signs suggestive of bilateral joint effusion and synovitis. A radiological examination revealed bilateral Kellgreen and Lawrence 4 tricompartmental gonarthrosis. A magnetic resonance imaging (MRI) study corroborated the diagnosis of anterior cruciate ligament rupture, advanced tricompartmental chondropathy, synovitis with joint effusion, and bilateral degenerative meniscus injury ( Fig. 1 ).

The patient was unaware he was an AKU carrier, and there was never any clinical suspicion of this diagnosis despite some typical manifestations, including dark pigmentation (gray and ochre) in the sclera, ear pinna, nose, and hands. The patient did not present other comorbidities, and basic preoperative tests did not raise suspicion.

Given the lack of AKU diagnosis, we opted for knee arthroscopy with ligament reconstruction, starting with the left one, which presented greater instability.

**Fig. 2:**
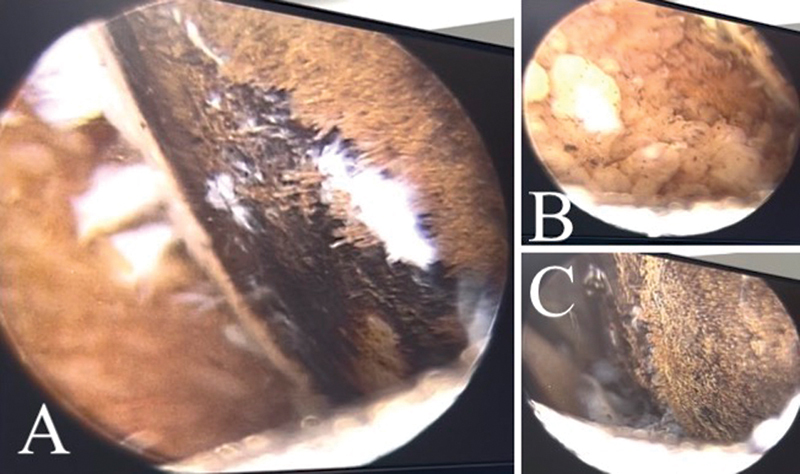
Knee arthroscopy showing advanced tricompartmental chondropathy, with a dark color suggesting intra-articular pigment deposits (ochronotic arthropathy). ( **A** ) Femoral condyle; ( **B** ) synovium; ( **C** ) femoral condyle.

During surgery, which began with the removal of the flexor graft, we observed an extremely dark gray and ocher pigmentation in the sartorius fascia and the gracilis and semitendinosus tendons. This finding led to knee arthroscopy, revealing synovitis, ligament injury, and advanced tricompartmental chondropathy with a dark color suggestive of pigment deposits in the articular cartilage, menisci, and ligaments ( Fig. 2 ). Since there was no diagnosis, we performed a synovectomy, collected a specimen for histopathological examination, and completed the procedure with intra-articular infiltration of hylan G-F 20 and 40 mg of triamcinolone hexacetonide ( Videos 1 and 2 ).

**Fig. 3:**
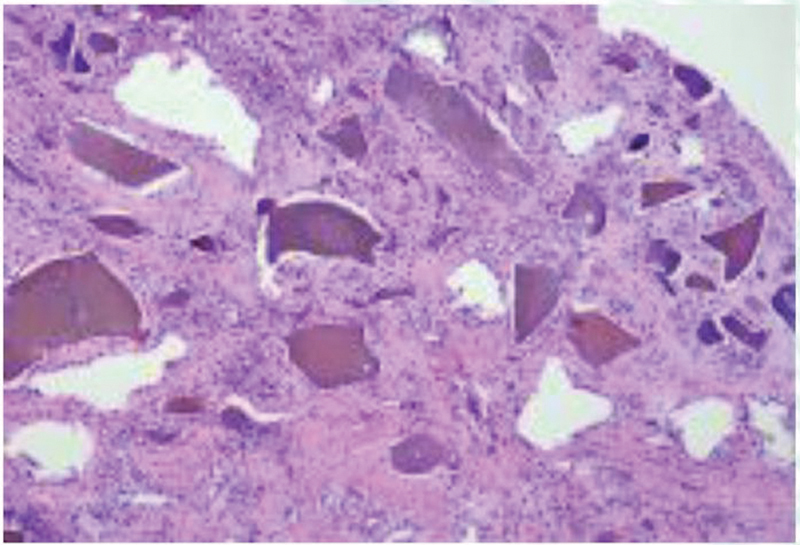
Histopathological examination of synovial tissue showing a chronic inflammatory process and pigment deposits.

**Fig. 4:**
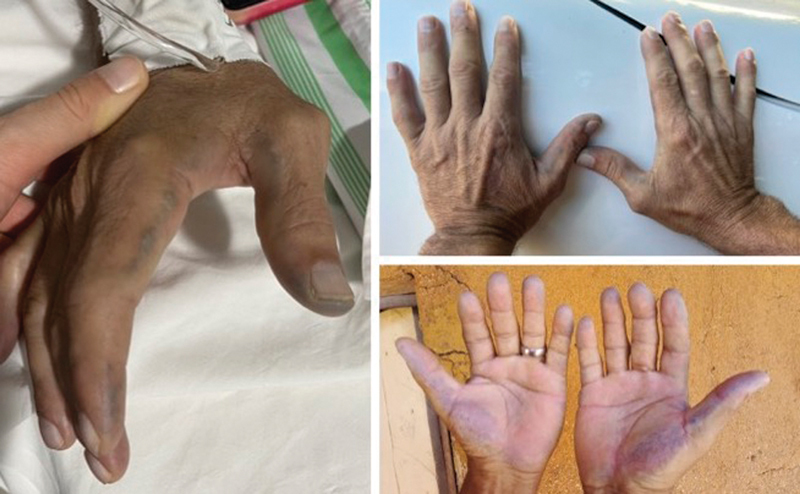
Patient's hands showing the characteristic skin pigmentation.

**Fig. 5:**
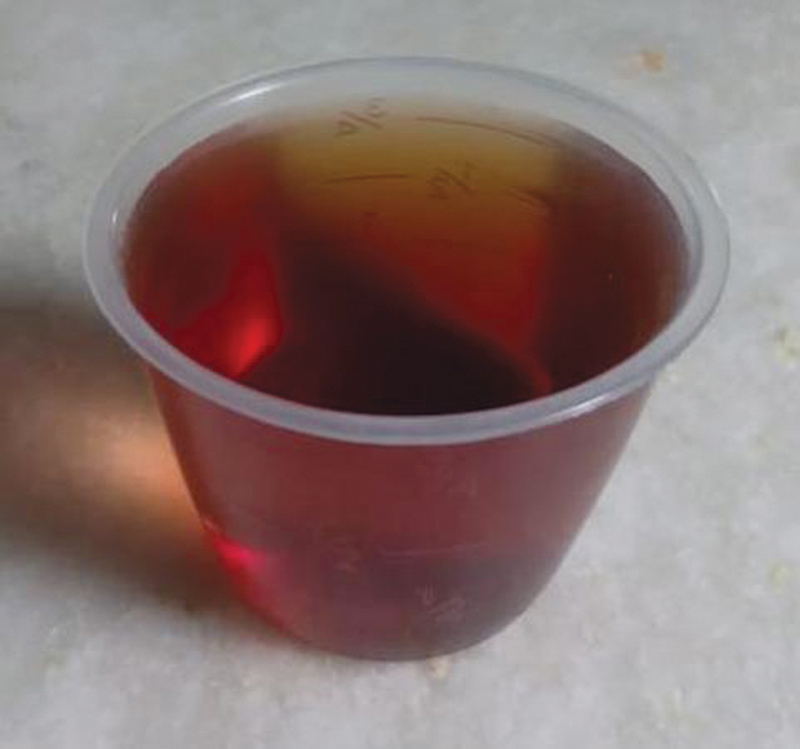
Dark-colored urine of the patient (homogentisic aciduria).

The histopathological examination reported “synovial tissue exhibiting a chronic inflammatory process and hemosiderin-like pigment deposits” ( Fig. 3 ). At the postoperative follow-up visit, we performed a more careful ectoscopic examination and anamnesis. There was dark pigmentation on the sclera, face, and hands. The patient stated that despite being asymptomatic, he had had dark urine since childhood, and that his two younger brothers had a similar condition ( Fig. 4 and 5 ). After reviewing the literature and considering the findings, we requested a test for homogentisic acid in the urine, which came back positive, confirming the diagnosis of AKU-related ochronotic arthropathy. An active search on the two younger brothers confirmed they were also AKU carriers. At the moment, the patient no longer complains of pain, but presents with residual instability. In agreement with the patient, we decided to continue with the conservative treatment (muscle strengthening and viscosupplementation) and contemplate an arthroplasty. Although his brothers report no complaints, they are under follow-up at our service.

## Discussion

The present case report shows that rare nosological entities can lead to misconduct. This fact highlights the significance of the present publication on ochronotic arthropathy/AKU.

Along with cystinuria, pentosuria, and albinism, AKU was one of four diseases described by Sir Archibald Edward Garrod as the result of the accumulation of metabolic intermediates due to metabolic deficiencies. 1 Moreover, AKU is one of the first human disorders to conform to the principles of recessive Mendelian inheritance. 5 Sir Garrod linked ochronosis with the accumulation of alkaptons in 1902, and his views on its mode of inheritance were summarized in a 1908 lecture given at the Royal College of Physicians, in London. The discovery of homogentisate 1,2 dioxygenase (HGD) deficiency as the enzymatic defect came 50 years later. 6 Sir Garrod pioneered the use of the expression *inborn errors of metabolism* to describe conditions caused by a failure in genetic biochemistry, also theorizing genes as responsible for enzyme synthesis.

The global incidence of AKU is low, with a frequency of 1 case in every 250 thousand to 1 million births. 7 The condition was identified in approximately 40 countries. 8 Investigation of affected families showed that they often reside in isolated communities, leading to the assumption of a higher incidence of the disease due to the founder effect, that is, the loss of genetic diversity resulting from genetic isolation. 9 Mutations in the *HGD* gene can lead to the development of ochronosis, but there is no specific correlation between the genotype and the phenotype. In other words, different mutations can result in the same clinical condition. 10 In Brazil, according to the Brazilian Society of Alkaptonuria (Sociedade Brasileira de Alcaptonúria, AKU Brasil, in Portuguese; www.akubrasil.com ), the number of carrier subjects is lower than 20.

Since there is no targeted treatment for AKU, therapy relies on symptomatic relief. In severe osteoarthritis, joint replacement surgery (arthroplasty) is the preferred treatment. 2 3 In the present report, we opted for the conservative treatment, with knee joint care and follow-up with viscosupplementation and muscle strengthening. When such conservative treatment proves no longer effective, we will assess the need for a total knee arthroplasty.
