# Supplementary material for: Ochronotic Arthropathy and Alkaptonuria (Ochronosis): Case Report
Source: Rev Bras Ortop (Sao Paulo). 2024 Apr 23;59(Suppl 2):e220–3. doi: 10.1055/s-0044-1779309 (PMC11679637; doi:10.1055/s-0044-1779309)
Supplement: Related texts

# Research article: Artropatia ocronótica e alcaptonúria (ocronose): Relato de caso

**Keywords:** alcaptonúria, artropatia, ocronose

## Abstract

A alcaptonúria ( *alkaptonuria* , AKU, em inglês) é uma condição genética rara em que a deficiência da enzima ácido homogentísico oxidase, produzida pelo fígado e pelos rins, interfere no metabolismo dos aminoácidos fenilalanina e tirosina. Embora possa não apresentar sintomas, a AKU pode levar à ocronose, um acúmulo anormal em tecidos do corpo de um pigmento chamado alcaptona. Com o passar do tempo, esse acúmulo de pigmento nas articulações pode levar a uma osteoartrose secundária conhecida como artropatia ocronótica, que é a forma mais incapacitante da doença. Devido à raridade da condição e à falta de conhecimento disseminado quanto a ela, relata-se um caso para descrever um diagnóstico de artropatia ocronótica de joelho que só foi identificado após a cirurgia. Devido à raridade dessa condição, sobretudo no Brasil, a descrição de casos ajudará na compreensão da epidemiologia nacional, assim como na divulgação de mais informações sobre a AKU e suas manifestações clínicas, sobretudo as osteoarticulares.

## Introdução

Alcaptonúria ( *alkaptonuria* , AKU, em inglês) é uma condição genética rara e autossômica recessiva resultante da falta da enzima ácido homogentísico oxidase, produzida pelo fígado e pelos rins, que desempenha um papel no metabolismo dos aminoácidos fenilalanina e tirosina. 1

A AKU pode não apresentar sintomas, ou pode levar ao desenvolvimento da ocronose, uma condição em que um polímero de ácido homogentísico é acumulado como pigmento alcaptona nos tecidos do corpo. 2 Cerca de metade dos portadores AKU desenvolvem ocronose, mais comumente homens, em uma proporção de 2:1, e geralmente após a idade de 40 anos. 2

A ocronose pode apresentar diversas manifestações clínicas importantes, incluindo artropatia, pigmentação ocular e cutânea, urina escura (acidúria homogentísica) e comprometimento cardiovascular. Com o passar dos anos, o acúmulo de pigmentos de ácido homogentísico nas articulações pode causar osteoartrose secundária, conhecida como artropatia ocronótica, que é a forma mais incapacitante da doença. 2 3

O diagnóstico da AKU é tipicamente baseado em achados clínicos, que são posteriormente confirmados pela detecção do ácido homogentísico na urina, em quantidades que podem chegar a cerca de 5g em um período de 24 horas. 2

Devido à raridade dessa condição, sobretudo no Brasil, a descrição de casos ajudará na compreensão da epidemiologia nacional, assim como na divulgação de mais informações sobre a AKU e suas manifestações clínicas, sobretudo as osteoarticulares.

## Relato de Caso

Um paciente do sexo masculino, de 46 anos, procurou o Serviço de Ortopedia com queixas álgicas e instabilidade bilateral de joelho. O paciente tinha o alinhamento dos membros inferiores preservado, com testes da gaveta anterior e *pivot-shift* positivos, dor à palpação na interlinha articular, e sinais clínicos sugestivos de derrame articular e sinovite, bilateralmente. Ao exame radiológico, observou-se gonartrose tricompartimental de grau III de Kellgreen e Lawrence 4 bilateralmente. O estudo de ressonância magnética (RM) corroborou o diagnóstico de ruptura de ligamento cruzado anterior, condropatia tricompartimental avançada e sinovite com derrame articular, além de lesão degenerativa dos meniscos, bilateralmente ( Fig. 1 ).

**Fig. 1:** Estudo de ressonância magnética de joelho esquerdo. ( **A** ) Corte sagital; ( **B** ) corte coronal; ( **C** ) corte axial.

O paciente não sabia que era portador de AKU, e em nenhum momento houve suspeita clínica desse diagnóstico, apesar de o paciente apresentar algumas manifestações, como pigmentação escura (cinza e ocre) nas escleras, no pavilhão auricular, no nariz e nas mãos. O paciente não apresentava outras comorbidades, e os exames básicos pré-operatórios não levantaram a suspeita.

Frente ao não diagnóstico de AKU, optamos pela artroscopia de joelho com reconstrução ligamentar, e iniciamos pelo esquerdo, do qual o paciente se queixava de maior instabilidade.

No ato operatório, iniciado pela retirada de enxerto dos flexores, observou-se pigmentação extremamente escura, cinza e ocre, na fáscia do sartório e nos tendões do grácil e do semitendíneo. Frente ao achado, foi iniciada uma artroscopia do joelho, na qual o inventário evidenciou sinovite, lesão ligamentar e condropatia tricompartimental avançada, com coloração escura sugestiva de depósito de pigmentos na cartilagem articular, nos meniscos e nos ligamentos ( Fig. 2 ). Como o caso não havia sido diagnosticado, foram realizadas sinovectomia, coleta de material para exame histopatológico, e o procedimento foi terminado com infiltração intrarticular com hilano G-F 20 e 40 mg de triancinolona hexacetonida ( Vídeos 1 e 2 ).

**Fig. 2:** Artroscopia do joelho evidenciando condropatia tricompartimental avançada, com coloração escura sugestiva de depósito intra-articular de pigmentos (artropatia ocronótica). ( **A** ) côndilo femoral; ( **B** ) sinóvia; ( **C** ) côndilo femoral.

**Vídeo 1** Artroscopia do joelho evidenciando condropatia tricompartimental avançada, com coloração escura sugestiva de depósito intra-articular de pigmentos (artropatia ocronótica).


**Vídeo 2** Artroscopia do joelho evidenciando condropatia tricompartimental avançada, com coloração escura sugestiva de depósito intra-articular de pigmentos (artropatia ocronótica).


O resultado do exame histopatológico veio como “tecido sinovial exibindo processo inflamatório crônico e depósito de pigmentos, semelhante a hemossiderina” ( Fig. 3 ). Na consulta de retorno pós-operatório, procedeu-se ao exame ectoscópico mais cuidadoso e à anamnese mais direcionada, observou-se a pigmentação escura nas escleras, face e mãos. O paciente afirmou que, apesar de assintomático, apresentava desde criança urina com aspecto escurecido, e que seus dois irmãos mais jovens tinham quadro semelhante ( Figs. 4 e 5 ). Após uma revisão da literatura e diante dos achados do caso, solicitamos a pesquisa de ácido homogentísico na urina, a qual veio positiva, e fechou-se o quadro de artropatia ocronótica por AKU. Fez-se uma busca ativa dos dois irmãos mencionados, e também confirmou-se que eram portadores de AKU. No momento, o paciente apresenta melhora das queixas álgicas, todavia, com instabilidade residualEm comum acordo com paciente, optou-se por seguir com o tratamento conservador (fortalecimento muscular e viscossuplementação), com possibilidade de artroplastia. Os irmãos não queixas, e estão em acompanhamento em nosso serviço.

**Fig. 3:** Exame histopatológico de tecido sinovial exibindo processo inflamatório crônico e depósito de pigmentos.

**Fig. 4:** Mãos do paciente evidenciando pigmentação cutânea característica.

**Fig. 5:** Urina com coloração escura do paciente (acidúria homogentísica).

## Discussão

Como visto no presente relato, entidades nosológicas raras podem levar a más condutas. Daí a importância da sua divulgação, como no caso da artropatia ocronótica/AKU.

A AKU foi uma das quatro doenças descritas por Sir Archibald Edward Garrod AKU, cistinúria, pentosúria e albinismo) como resultantes do acúmulo de intermediários metabólicos devido a deficiências metabólicas, 1 e um dos primeiros distúrbios em humanos encontrados em conformidade com os princípios da herança recessiva mendeliana. 5 Sir Archibald vinculou a ocronose ao acúmulo de alcaptanos em 1902, e suas opiniões sobre o modo de herança foram resumidos em uma palestra proferida em 1908 no Royal College of Physicians, em Londres. Somente após 50 anos, o defeito enzimático foi atribuído à deficiência de homogentisato 1,2 dioxigenase (HGD). 6 Sir Archibald foi pioneiro no uso da expressão *erros inatos do metabolismo* para descrever as patologias ocasionadas por uma falha na bioquímica genética, e também sugeriu a teoria de que os genes seriam responsáveis pela síntese das enzimas.

A incidência global da AKU é rara, com uma frequência de 1 caso em cada 250 mil a 1 milhão de nascimentos, 7 e casos já foram identificados em cerca de 40 países. 8 A investigação das famílias afetadas mostrou que elas frequentemente residem em comunidades isoladas, o que leva à suposição de que a alta incidência da doença é principalmente devida ao efeito fundador, ou seja, à perda de diversidade genética como resultado do isolamento genético. 9 As mutações no gene *HGD* podem levar ao desenvolvimento de ocronose, mas não há uma correlação específica entre o genótipo e o fenótipo, ou seja, várias mutações diferentes podem resultar na mesma condição clínica. 10 No Brasil, segundo a Sociedade Brasileira de Alcaptonúria (AKU Brasil, www.akubrasil.com ), o número de indivíduos portadores não chega a 20.

Como não há um tratamento direcionado para a AKU, a terapia é baseada no alívio dos sintomas. Em situações graves de osteoartrite, a cirurgia de substituição articular (artroplastia) é a opção de tratamento preferencial. 2 3 No presente relato, optamos pelo tratamento conservador, com toalete articular do joelho e seguimento com viscossuplementação e fortalecimento muscular. Quando tal tratamento conservador não se mostrar mais eficaz, avaliaremos a necessidade de proceder à artroplastia total do joelho.
